# 2-Bromo-*N*-(dibenzyl­carbamothioyl)benzamide

**DOI:** 10.1107/S1600536811014711

**Published:** 2011-04-29

**Authors:** Mohd Faizal Md Nasir, Ibrahim N. Hassan, Wan Ramli Wan Daud, Bohari M. Yamin, Mohammad B. Kassim

**Affiliations:** a Fuel Cell Institute, Universiti Kebangsaan Malaysia, UKM 43600 Bangi Selangor, Malaysia; b Department of Chemical and Process Engineering, Faculty of Engineering, Universiti Kebangsaan Malaysia, UKM 43600 Bangi Selangor, Malaysia; c School of Chemical Sciences and Food Technology, Faculty of Science and Technology, Universiti Kebangsaan Malaysia, UKM 43600 Bangi Selangor, Malaysia

## Abstract

The 2-bromo­benzoyl group in the title compound, C_22_H_19_BrN_2_OS, adopts an *E* conformation with respect to the thiono S atom across the N—C bond. In the crystal structure, the mol­ecule is stablized by N—H⋯O inter­molecular hydrogen bonds, forming a one-dimensional chain along the *b* axis.

## Related literature

For related structures, see: Yamin & Hassan (2004 ▶); Hassan *et al.* (2008*a* ▶,*b* ▶,*c* ▶, 2009 ▶). For the synthesis, see: Hassan *et al.* (2008*a* ▶). For reference bond distances, see: Allen *et al.* (2004 ▶).
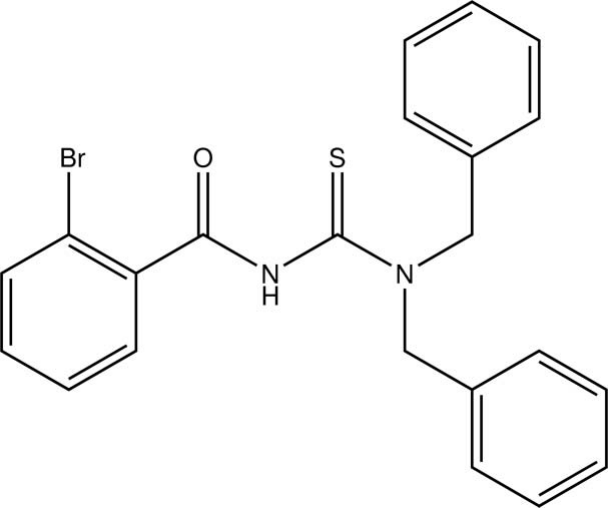


## Experimental

### 

#### Crystal data

C_22_H_19_BrN_2_OS
*M* *_r_* = 439.36
Tetragonal, 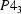

*a* = 12.2833 (16) Å
*c* = 14.002 (4) Å
*V* = 2112.6 (7) Å^3^
*Z* = 4
Mo *K*α radiation
μ = 2.06 mm^−1^
*T* = 273 K
0.35 × 0.31 × 0.23 mm

#### Data collection

Bruker SMART APEX CCD area-detector diffractometer
Absorption correction: multi-scan (*SADABS*; Bruker, 2000 ▶) *T*_min_ = 0.533, *T*_max_ = 0.649
15683 measured reflections
5217 independent reflections
2506 reflections with *I* > 2σ(*I*)
*R*_int_ = 0.064

#### Refinement

*R*[*F* ^2^ > 2σ(*F* ^2^)] = 0.050
*wR*(*F* ^2^) = 0.118
*S* = 0.93
5217 reflections
244 parameters
1 restraint
H-atom parameters constrained
Δρ_max_ = 0.57 e Å^−3^
Δρ_min_ = −0.20 e Å^−3^
Absolute structure: Flack (1983 ▶), with 2474 Friedel pairs
Flack parameter: −0.001 (11)

### 

Data collection: *SMART* (Bruker, 2000 ▶); cell refinement: *SAINT* (Bruker, 2000 ▶); data reduction: *SAINT*; program(s) used to solve structure: *SHELXS97* (Sheldrick, 2008 ▶); program(s) used to refine structure: *SHELXL97* (Sheldrick, 2008 ▶); molecular graphics: *ORTEPIII* (Burnett & Johnson, 1996 ▶), *ORTEP-3 for Windows* (Farrugia, 1997 ▶) and *PLATON* (Spek, 2009 ▶); software used to prepare material for publication: *SHELXTL* (Sheldrick, 2008 ▶), *PARST* (Nardelli, 1995 ▶) and *PLATON*.

## Supplementary Material

Crystal structure: contains datablocks global, I. DOI: 10.1107/S1600536811014711/dn2677sup1.cif

Structure factors: contains datablocks I. DOI: 10.1107/S1600536811014711/dn2677Isup2.hkl

Additional supplementary materials:  crystallographic information; 3D view; checkCIF report

## Figures and Tables

**Table 1:** Hydrogen-bond geometry (Å, °)

*D*—H⋯*A*	*D*—H	H⋯*A*	*D*⋯*A*	*D*—H⋯*A*
N1—H1*A*⋯Br1	0.86	2.79	3.220 (3)	113
N1—H1*A*⋯O1^i^	0.86	2.20	2.903 (4)	139

Symmetry code: (i) 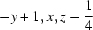.

**Fig. 1.**
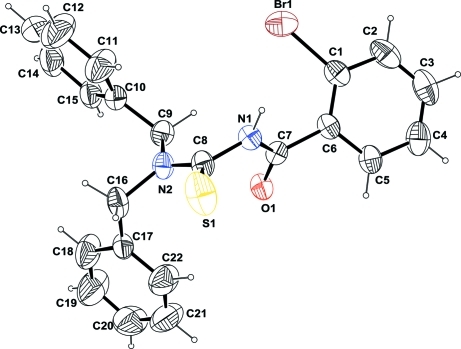
The molecular structure of (I), with the atoms labeling scheme. Displacement ellipsoids are drawn at the 30% probability level. H atoms are represented as small spheres of arbitrary radii.

**Fig. 2.**
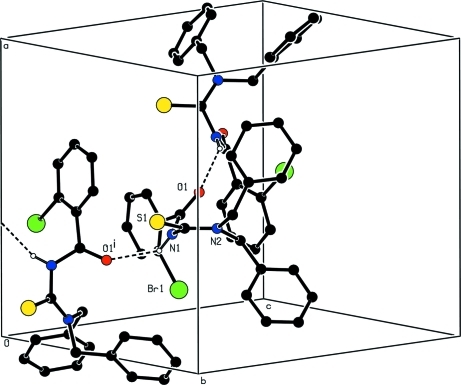
Partial packing view of (I) showing the formation of the chain through N—H···O hydrogen bondings. H bonds are shown as dashed lines. [Symmetry code: (i) -y + 1, x, z - 1/4]

## supplementary crystallographic information

### Comment

The title compound, I, is a thiourea derivative of dibenzylamine analogous to our previous reported, ethyl-2-(3-benzoylthioureido)acetate (Hassan *et al.*, 2008*a*), propyl-2-(3-benzoylthioureido)acetate (Hassan *et al.*, 2008*b*), butyl-2-(3-benzoylthioureido)acetate (Hassan *et al.*, 2008*c*) and 1-(2-morpholinoethyl)-3-(3-phenylacryloyl)thiourea (Yamin & Hassan, 2004). The molecule has the 2-bromobenzoyl group adopting an *E* conformation, with respect to the thiono S atom across the N1—C8 bond, whereas both the phenyl ring of the dibenzylamine group adopt *E* and *Z* conformation relative to the S atom across the N2—C8 bond (Fig. 1). The phenyl ring, (C1–C6), and the thiourea fragment, (S1/N1/N2/C8), are essentially planar and the dihedral angle between them is 72.9 (2)°. The bond lengths and angles in the molecules are in normal ranges (Allen *et al.*, 1987).

Both phenyl rings, [C10/C11/C12/C13/C14/C15] and [C17/C18/C19/C20/C21/C22] are essentially planar and they are twisted to each other by a dihedral angle of 22.4 (4)°. There is weak intramolecular hydrogen bond, N1—H1A···Br1 (Table 1). As a result, one pseudo-six-membered ring (N1/H1A/Br1/C1/C6/C7) is formed. The intermolecular N1—H1A···O1 hydrogen bonds (Table 1,) links the molecules into a chain parallel to the *b* axis (Fig. 2).

### Experimental

The title compound was synthesized according to a previously reported compound (Hassan *et al.*, 2008*a*). A colourless crystal, suitable for X-ray crystallography, was obtained by a slow evaporation from methanolic solution at room temperature (yield 83%).

### Refinement

H atoms of both C and N atoms were positioned geometrically and allowed to ride on their parent atoms, with *U*_iso_ = 1.2*U*_eq_(C) for aromatic 0.93 Å, *U*_iso_ = 1.2*U*_eq_ (C) for CH_2_ 0.97 Å, *U*_iso_ = 1.2*U*_eq_ (N) for N—H 0.86 Å.

### Crystal data

**Table:**

C_22_H_19_BrN_2_OS	*D*_x_ = 1.381 Mg m^−^^3^
*M**_r_* = 439.36	Mo *K*α radiation, λ = 0.71073 Å
Tetragonal, *P*4_3_	Cell parameters from 2531 reflections
Hall symbol: P 4cw	θ = 1.7–28.4°
*a* = 12.2833 (16) Å	µ = 2.06 mm^−^^1^
*c* = 14.002 (4) Å	*T* = 273 K
*V* = 2112.6 (7) Å^3^	Block, colourless
*Z* = 4	0.35 × 0.31 × 0.23 mm
*F*(000) = 896	

### Data collection

**Table:**

Bruker SMART APEX CCD area-detector diffractometer	5217 independent reflections
Radiation source: fine-focus sealed tube	2506 reflections with *I* > 2σ(*I*)
graphite	*R*_int_ = 0.064
ω scans	θ_max_ = 28.4°, θ_min_ = 1.7°
Absorption correction: multi-scan (*SADABS*; Sheldrick, 2000)	*h* = −12→16
*T*_min_ = 0.533, *T*_max_ = 0.649	*k* = −14→16
15683 measured reflections	*l* = −18→18

### Refinement

**Table:**

Refinement on *F*^2^	Secondary atom site location: difference Fourier map
Least-squares matrix: full	Hydrogen site location: inferred from neighbouring sites
*R*[*F*^2^ > 2σ(*F*^2^)] = 0.050	H-atom parameters constrained
*wR*(*F*^2^) = 0.118	*w* = 1/[σ^2^(*F*_o_^2^) + (0.0418*P*)^2^] where *P* = (*F*_o_^2^ + 2*F*_c_^2^)/3
*S* = 0.93	(Δ/σ)_max_ < 0.001
5217 reflections	Δρ_max_ = 0.57 e Å^−^^3^
244 parameters	Δρ_min_ = −0.20 e Å^−^^3^
1 restraint	Absolute structure: Flack (1983), with 2474 Friedel pairs
Primary atom site location: structure-invariant direct methods	Flack parameter: −0.001 (11)

### Special details

**Table:**

Geometry. All esds (except the esd in the dihedral angle between two l.s. planes) are estimated using the full covariance matrix. The cell esds are taken into account individually in the estimation of esds in distances, angles and torsion angles; correlations between esds in cell parameters are only used when they are defined by crystal symmetry. An approximate (isotropic) treatment of cell esds is used for estimating esds involving l.s. planes.
Refinement. Refinement of *F*^2^ against ALL reflections. The weighted *R*-factor *wR* and goodness of fit *S* are based on *F*^2^, conventional *R*-factors *R* are based on *F*, with *F* set to zero for negative *F*^2^. The threshold expression of *F*^2^ > σ(*F*^2^) is used only for calculating *R*-factors(gt) *etc*. and is not relevant to the choice of reflections for refinement. *R*-factors based on *F*^2^ are statistically about twice as large as those based on *F*, and *R*-factors based on ALL data will be even larger.

### Fractional atomic coordinates and isotropic or equivalent isotropic displacement parameters (Å^2^)

**Table:**

	*x*	*y*	*z*	*U*_iso_*/*U*_eq_	
Br1	0.20156 (4)	0.59332 (4)	0.23207 (5)	0.0975 (2)	
S1	0.48743 (15)	0.81061 (10)	−0.01270 (10)	0.1136 (5)	
O1	0.5373 (2)	0.6765 (2)	0.2480 (2)	0.0656 (7)	
N1	0.4138 (2)	0.6950 (2)	0.1306 (2)	0.0552 (8)	
H1A	0.3584	0.6663	0.1027	0.066*	
N2	0.4512 (3)	0.8761 (3)	0.1662 (2)	0.0642 (9)	
C1	0.3189 (3)	0.4915 (3)	0.2306 (3)	0.0685 (11)	
C2	0.2938 (5)	0.3838 (5)	0.2467 (4)	0.0973 (16)	
H2A	0.2218	0.3628	0.2562	0.117*	
C3	0.3751 (6)	0.3079 (4)	0.2485 (4)	0.1067 (18)	
H3A	0.3579	0.2349	0.2578	0.128*	
C4	0.4787 (5)	0.3375 (4)	0.2371 (5)	0.0959 (15)	
H4A	0.5333	0.2851	0.2400	0.115*	
C5	0.5061 (4)	0.4451 (3)	0.2210 (3)	0.0684 (11)	
H5A	0.5786	0.4647	0.2126	0.082*	
C6	0.4249 (3)	0.5240 (3)	0.2174 (3)	0.0567 (10)	
C7	0.4632 (3)	0.6395 (3)	0.2022 (3)	0.0517 (10)	
C8	0.4501 (4)	0.7972 (3)	0.1011 (3)	0.0638 (11)	
C9	0.3979 (4)	0.8690 (3)	0.2597 (3)	0.0694 (12)	
H9A	0.4523	0.8763	0.3095	0.083*	
H9B	0.3645	0.7979	0.2663	0.083*	
C10	0.3114 (4)	0.9563 (4)	0.2731 (4)	0.0733 (13)	
C11	0.2355 (5)	0.9757 (5)	0.2031 (5)	0.120 (2)	
H11A	0.2383	0.9383	0.1454	0.144*	
C12	0.1542 (6)	1.0525 (7)	0.2202 (8)	0.156 (3)	
H12A	0.1035	1.0672	0.1726	0.187*	
C13	0.1477 (6)	1.1055 (6)	0.3037 (9)	0.137 (3)	
H13A	0.0923	1.1556	0.3145	0.164*	
C14	0.2215 (7)	1.0855 (5)	0.3709 (6)	0.116 (2)	
H14A	0.2171	1.1222	0.4289	0.139*	
C15	0.3049 (5)	1.0111 (4)	0.3565 (4)	0.0841 (14)	
H15A	0.3562	0.9991	0.4041	0.101*	
C16	0.5113 (4)	0.9773 (3)	0.1497 (4)	0.0799 (13)	
H16A	0.4655	1.0387	0.1664	0.096*	
H16B	0.5292	0.9831	0.0824	0.096*	
C17	0.6145 (4)	0.9818 (3)	0.2075 (4)	0.0722 (13)	
C18	0.6297 (6)	1.0599 (5)	0.2746 (5)	0.117 (2)	
H18A	0.5758	1.1119	0.2839	0.140*	
C19	0.7213 (7)	1.0642 (6)	0.3286 (7)	0.161 (4)	
H19A	0.7279	1.1164	0.3763	0.193*	
C20	0.8018 (6)	0.9934 (7)	0.3131 (6)	0.134 (3)	
H20A	0.8672	1.0007	0.3459	0.160*	
C21	0.7890 (5)	0.9132 (6)	0.2515 (7)	0.132 (3)	
H21A	0.8432	0.8609	0.2443	0.158*	
C22	0.6946 (5)	0.9072 (5)	0.1976 (4)	0.1103 (19)	
H22A	0.6861	0.8508	0.1539	0.132*	

### Atomic displacement parameters (Å^2^)

**Table:**

	*U*^11^	*U*^22^	*U*^33^	*U*^12^	*U*^13^	*U*^23^
Br1	0.0739 (3)	0.1215 (4)	0.0970 (4)	−0.0048 (3)	0.0148 (3)	0.0028 (4)
S1	0.1990 (16)	0.0888 (8)	0.0531 (7)	−0.0186 (9)	0.0167 (10)	0.0149 (8)
O1	0.0769 (17)	0.0579 (16)	0.0620 (19)	−0.0059 (14)	−0.0219 (16)	0.0042 (14)
N1	0.064 (2)	0.056 (2)	0.0457 (18)	−0.0031 (16)	−0.0102 (15)	0.0076 (14)
N2	0.082 (2)	0.050 (2)	0.061 (2)	0.0049 (18)	−0.0021 (18)	0.0128 (18)
C1	0.083 (3)	0.072 (3)	0.051 (2)	−0.016 (2)	0.003 (3)	0.007 (2)
C2	0.108 (4)	0.097 (4)	0.087 (4)	−0.042 (4)	0.011 (3)	0.011 (3)
C3	0.156 (6)	0.066 (3)	0.098 (4)	−0.026 (4)	−0.007 (4)	0.014 (3)
C4	0.134 (5)	0.063 (3)	0.090 (4)	0.012 (3)	−0.004 (4)	0.019 (3)
C5	0.092 (3)	0.057 (2)	0.056 (3)	0.000 (2)	−0.005 (2)	0.013 (2)
C6	0.075 (3)	0.055 (2)	0.041 (2)	−0.009 (2)	−0.0036 (19)	0.0091 (18)
C7	0.060 (2)	0.056 (2)	0.039 (2)	0.007 (2)	−0.0019 (18)	0.0025 (17)
C8	0.079 (3)	0.054 (3)	0.058 (3)	0.001 (2)	−0.009 (2)	0.013 (2)
C9	0.076 (3)	0.065 (3)	0.067 (3)	0.006 (2)	−0.003 (2)	0.004 (2)
C10	0.070 (3)	0.056 (3)	0.094 (4)	0.001 (2)	−0.003 (3)	−0.004 (2)
C11	0.110 (4)	0.113 (4)	0.135 (6)	0.034 (4)	−0.056 (4)	−0.034 (4)
C12	0.117 (5)	0.139 (6)	0.212 (10)	0.046 (5)	−0.072 (6)	−0.031 (7)
C13	0.082 (5)	0.090 (5)	0.237 (10)	0.007 (4)	0.039 (6)	−0.006 (6)
C14	0.143 (6)	0.065 (4)	0.140 (6)	−0.003 (4)	0.042 (5)	−0.017 (4)
C15	0.099 (4)	0.058 (3)	0.095 (4)	−0.002 (3)	0.009 (3)	−0.009 (3)
C16	0.109 (4)	0.049 (3)	0.082 (3)	−0.004 (3)	0.005 (3)	0.013 (2)
C17	0.080 (3)	0.048 (2)	0.089 (4)	0.000 (2)	0.011 (3)	−0.002 (2)
C18	0.129 (5)	0.079 (4)	0.143 (5)	0.021 (4)	−0.033 (4)	−0.041 (4)
C19	0.125 (6)	0.128 (5)	0.229 (10)	0.021 (5)	−0.057 (6)	−0.094 (6)
C20	0.095 (5)	0.148 (6)	0.159 (7)	−0.013 (5)	−0.022 (4)	−0.042 (5)
C21	0.087 (4)	0.143 (6)	0.166 (7)	0.027 (4)	0.004 (5)	−0.039 (6)
C22	0.105 (4)	0.110 (4)	0.116 (5)	0.013 (4)	0.007 (4)	−0.041 (3)

### Geometric parameters (Å, °)

**Table:**

Br1—C1	1.909 (4)	C10—C11	1.372 (7)
S1—C8	1.666 (4)	C11—C12	1.394 (10)
O1—C7	1.203 (4)	C11—H11A	0.9300
N1—C7	1.356 (5)	C12—C13	1.341 (11)
N1—C8	1.396 (5)	C12—H12A	0.9300
N1—H1A	0.8602	C13—C14	1.329 (11)
N2—C8	1.330 (5)	C13—H13A	0.9300
N2—C16	1.464 (6)	C14—C15	1.388 (8)
N2—C9	1.467 (5)	C14—H14A	0.9300
C1—C6	1.375 (5)	C15—H15A	0.9300
C1—C2	1.376 (6)	C16—C17	1.505 (7)
C2—C3	1.367 (8)	C16—H16A	0.9700
C2—H2A	0.9300	C16—H16B	0.9700
C3—C4	1.334 (8)	C17—C22	1.352 (7)
C3—H3A	0.9300	C17—C18	1.355 (7)
C4—C5	1.382 (6)	C18—C19	1.356 (9)
C4—H4A	0.9300	C18—H18A	0.9300
C5—C6	1.392 (5)	C19—C20	1.334 (9)
C5—H5A	0.9300	C19—H19A	0.9300
C6—C7	1.509 (5)	C20—C21	1.319 (10)
C9—C10	1.521 (6)	C20—H20A	0.9300
C9—H9A	0.9700	C21—C22	1.386 (9)
C9—H9B	0.9700	C21—H21A	0.9300
C10—C15	1.350 (7)	C22—H22A	0.9300
			
C7—N1—C8	121.9 (3)	C10—C11—C12	118.7 (6)
C7—N1—H1A	119.0	C10—C11—H11A	120.6
C8—N1—H1A	119.2	C12—C11—H11A	120.6
C8—N2—C16	121.0 (4)	C13—C12—C11	121.4 (7)
C8—N2—C9	124.3 (3)	C13—C12—H12A	119.3
C16—N2—C9	114.6 (4)	C11—C12—H12A	119.3
C6—C1—C2	120.9 (4)	C14—C13—C12	119.1 (7)
C6—C1—Br1	121.7 (3)	C14—C13—H13A	120.4
C2—C1—Br1	117.3 (4)	C12—C13—H13A	120.4
C3—C2—C1	119.7 (5)	C13—C14—C15	121.4 (7)
C3—C2—H2A	120.2	C13—C14—H14A	119.3
C1—C2—H2A	120.2	C15—C14—H14A	119.3
C4—C3—C2	120.6 (5)	C10—C15—C14	119.9 (6)
C4—C3—H3A	119.7	C10—C15—H15A	120.1
C2—C3—H3A	119.7	C14—C15—H15A	120.1
C3—C4—C5	120.8 (5)	N2—C16—C17	111.8 (4)
C3—C4—H4A	119.6	N2—C16—H16A	109.3
C5—C4—H4A	119.6	C17—C16—H16A	109.3
C4—C5—C6	119.9 (5)	N2—C16—H16B	109.3
C4—C5—H5A	120.1	C17—C16—H16B	109.3
C6—C5—H5A	120.1	H16A—C16—H16B	107.9
C1—C6—C5	118.1 (4)	C22—C17—C18	116.8 (5)
C1—C6—C7	126.0 (4)	C22—C17—C16	122.2 (5)
C5—C6—C7	115.9 (4)	C18—C17—C16	121.0 (5)
O1—C7—N1	122.9 (3)	C17—C18—C19	121.9 (6)
O1—C7—C6	121.1 (3)	C17—C18—H18A	119.1
N1—C7—C6	115.9 (3)	C19—C18—H18A	119.1
N2—C8—N1	117.1 (3)	C20—C19—C18	119.9 (7)
N2—C8—S1	125.5 (3)	C20—C19—H19A	120.0
N1—C8—S1	117.4 (3)	C18—C19—H19A	120.0
N2—C9—C10	112.3 (4)	C21—C20—C19	120.3 (7)
N2—C9—H9A	109.1	C21—C20—H20A	119.8
C10—C9—H9A	109.1	C19—C20—H20A	119.8
N2—C9—H9B	109.1	C20—C21—C22	119.7 (6)
C10—C9—H9B	109.1	C20—C21—H21A	120.2
H9A—C9—H9B	107.9	C22—C21—H21A	120.2
C15—C10—C11	119.4 (5)	C17—C22—C21	121.1 (5)
C15—C10—C9	119.9 (5)	C17—C22—H22A	119.4
C11—C10—C9	120.6 (5)	C21—C22—H22A	119.4

### Hydrogen-bond geometry (Å, °)

**Table:**

*D*—H···*A*	*D*—H	H···*A*	*D*···*A*	*D*—H···*A*
N1—H1A···Br1	0.86	2.79	3.220 (3)	113.
N1—H1A···O1^i^	0.86	2.20	2.903 (4)	139.

Symmetry codes: (i) −*y*+1, *x*, *z*−1/4.
